# The Interactive Effects between Drought and Air Pollutants on Children’s Upper Respiratory Tract Infection: A Time-Series Analysis in Gansu, China

**DOI:** 10.3390/ijerph20031959

**Published:** 2023-01-20

**Authors:** Yanlin Li, Jianyun Sun, Ruoyi Lei, Jie Zheng, Xiaoyu Tian, Baode Xue, Bin Luo

**Affiliations:** 1 Institute of Occupational Health and Environmental Health, School of Public Health, Lanzhou University, Lanzhou 730000, China; 2 Gansu Provincial Centre for Diseases Prevention and Control, Lanzhou 730000, China; 3 Shanghai Key Laboratory of Meteorology and Health, Shanghai Meteorological Bureau, Shanghai 200030, China; 4 Shanghai Typhoon Institute, China Meteorological Administration, Shanghai 200030, China

**Keywords:** drought, URTI, SPI, air pollutants, GAM, DLNM

## Abstract

As a destructive and economic disaster in the world, drought shows an increasing trend under the continuous global climate change and adverse health effects have been reported. The interactive effects between drought and air pollutants, which may also be harmful to respiratory systems, remain to be discussed. We built the generalized additive model (GAM) and distributed lag nonlinear model (DLNM) to estimate the effects of drought and air pollutants on daily upper respiratory infections (URTI) outpatient visits among children under 6 in three cities of Gansu province. The Standardized Precipitation Index (SPI) based on monthly precipitation (SPI-1) was used as an indicator of drought. A non-stratified model was established to explore the interaction effect of SPI-1 and air pollutants. We illustrated the number of daily pediatric URTI outpatient visits increased with the decrease in SPI-1. The interactive effects between air pollutants and the number of daily pediatric URTIs were significant. According to the non-stratified model, we revealed highly polluted and drought environments had the most significant impact on URTI in children. The occurrence of drought and air pollutants increased URTI in children and exhibited a significant interactive effect.

## 1. Introduction

In the context of global climate change, extreme weather events (EWEs) such as heat waves, floods, droughts, cold spells, storms, and other EWEs occur continually [1,2], causing direct or indirect adverse effects on human health [3,4,5]. As a vulnerable group, children are more environmentally sensitive due to their physical and mental immaturity [6]. Numerous studies have linked EWEs to children’s respiratory diseases, mental diseases, allergic diseases, and malnutrition [1,7,8,9,10,11]. Under continuous global climate change, children’s health will be further challenged by the increasing EWEs, and environmental and socioeconomic pressure [12].

Among EWEs, drought is a widespread climatic phenomenon with high frequency, wide influence, and long duration, which is one of the most destructive and economical disasters in the world [13]. Climate change exacerbates drought events, which increased exposure to wildfires and environmental pollutants, and threatened water security, sanitation, and food production [14,15]. Climate change may lead to changes in precipitation patterns and the probability of extreme dry events, affecting airborne particulate matter (PM) and land desertification, and exacerbating dust events [16]. An Asian dust storm (ADS) is a seasonal meteorological phenomenon, which originates in the deserts of Mongolia and northern China and spreads eastward along the mid-latitude westerly winds [17,18]. During long-distance transport, the ADS mixes with a variety of pollutants, causing damage to the environment and the human body [19], especially adverse health effects on the respiratory system [20,21]. A research study in the Brazilian Amazon showed a significant increase in the number of children under five hospitalized for respiratory diseases in highly arid cities because drought conditions were associated with fire incidence, aerosol emissions, and degradation in air quality [22]. Recent studies have indicated that the occurrence of drought harms people’s psychological and physical health, but direct evidence from research on drought-related adverse health effects in children is still relatively rare [23,24,25].

Air pollutants are closely related to human health, and the effects on the respiratory system are more direct and significant [26,27]. A previous study based on 252 cities in China found that short-term exposure to PM_2.5_ and O_3_ was associated with an increased risk of hospitalization for respiratory diseases [28]. Even at low concentrations, air pollutants still had negative effects on the children’s respiratory system [29]. As for children, a multi-city study indicated that short-term exposure to air pollutants was associated with increased respiratory disease hospitalization [30]. The etiology of upper respiratory tract infections (URTI) is complex, environmental factors such as meteorological variants are also considered as an important factor apart from pathogen infection [21,31]. However, no study has reported the interactive effects of drought and air pollutants on child URTI.

A variety of viruses and bacteria can cause URTI, involving the nose, sinuses, pharynx, larynx, and respiratory tract [32]. URTI is one of the most common types of disease in outpatients, the incidence of which is higher in winter and spring than in other seasons [33]. Children are immunocompromised and at high risk of URTI, who can occur multiple times within a year [34]. To explore the effects of the interaction between drought and air pollutants on child URTI, we applied the Standardized Precipitation Index (SPI) as a parameter to describe drought conditions in Gansu province and conducted a time-series study to evaluate the effects on child URTI outpatient visits.

## 2. Methods

### 2.1. Study Area and Data Collection

Gansu province is located in northwest China, at the intersection of three plateaus, with long and narrow topography and various climate types. Most of Gansu province has a dry climate, with arid and semi-arid areas accounting for 75% of the total area [35]. In this study, we chose the central urban areas of three major cities in Gansu province as the study area (Lanzhou, Tianshui, and Zhangye city). Daily data on outpatient visits for pediatric URTI between 1 January 2015 and 31 December 2018 were collected from Gansu Provincial Maternity and Children-care Hospital (the largest pediatric hospital in Lanzhou), the First Hospital of Tianshui City, and People’s Hospital of Zhangye City, all of which were the biggest hospital in each city. The outpatient visit data for URTI (J00-J06) of children under 6 were screened by the International Classification of Diseases (ICD-10) [36].

Data on daily air pollutants, including PM (PM_2.5_ and PM_10_), nitrogen dioxide (NO_2_), sulfur dioxide (SO_2_), carbon monoxide (CO), and ozone (O_3_), were collected from a website of the Data Center for the Ministry of Ecology and Environment of the People’s Republic of China (http://datacenter.mee.gov.cn/, accessed on 1 December 2022).

To obtain meteorological data in each city, we chose the closest weather monitoring stations to each hospital to collect the data of daily temperature, relative humidity, and precipitation data of Tianshui and Zhangye city from the National Greenhouse Data Sharing Platform (http://data.sheshiyuanyi.com/WeatherData/, accessed on 1 December 2022). The daily meteorological data of Lanzhou were obtained from the Lanzhou Meteorological Bureau.

### 2.2. Drought Index

Drought is a natural disaster that occurs quietly and is caused by lower precipitation than normal [18,37]. The SPI is an indicator that characterizes precipitation over a certain period and is suitable for monitoring and assessing drought at different time scales in different regions [38]. In this study, we used the weekly SPI which was calculated by 1 month’s accumulation of precipitation (SPI-1) as the drought index for each city. The SPI-1 was calculated by the latest SPI program (http://drought.unl.edu/droughtmonitoring/SPI/SPIProgram.aspx, accessed on 4 December 2022). According to Grades of Meteorological Drought (GB/T 20481-2017) of China, when SPI-1 is equal to or less than −0.5, it indicates a drought event in the area. In this study, the severity of the drought period in three cities was classified into four grades (“no drought”, “light drought”, “moderate drought”, and “severe drought”) according to SPI-1 values.

### 2.3. Statistical Analysis

#### 2.3.1. GAM Model

Spearman correlation analyses were conducted among meteorological factors, air pollutants, and SPI-1. To estimate the association between drought and pediatric URTI, a time-series quasi-Poisson distribution generalized additive model (GAM) was fitted. Long-term time trends and meteorological factors were adjusted by the natural cubic smooth function in the model. The model was fitted by R software (version 4.1.3) and the equation was as follows:

(1) $$Log\left[E\left(Y_{k}\right)\right]=\alpha +\beta SPI-1_{k}+ns\left(time,\;\frac{7}{y}\right)+ns\left(AT,\;6\right)+ns\left(RH,\;6\right)+DOW+holiday$$

In the equation, *Y_k_* is the observed outpatient visits on day k, and *E*[(*Y_k_*)] is the expected outpatient visits on day k; *α* is the intercept, and *β* is the regression coefficients; *ns*( ) is the natural cubic spline; *time* is the days of calendar time on day *k*, and the 7 degrees of freedom (*df*) per year (*y*) are used to adjust long time trends; *AT* and *RH* are the 3-day moving average mean ambient temperature and relative humidity, which are used to control the potential nonlinear effects, and 6 are the *df*; *DOW* is the day of the week which is a categorical variate; and the *holiday* is a binary variate denoting public holidays in China. Due to the strong correlation between temperature and air pollutants, we did not adjust the air pollutants in the model. The values of *df* were determined by the relevant literature and the Akaike information criterion (AIC).

#### 2.3.2. DLNM Model

The distributed lag nonlinear model (DLNM) with a quasi-Poisson distribution was built to estimate the association between six different air pollutants (PM_2.5_, PM_10_, NO_2_, SO_2_, CO, and O_3_) and outpatient visits of pediatric URTI in each city. Due to the city-specific effect, we applied a multivariate meta-analysis fitted by the “mvmeta” package to combine the overall effects which were obtained from three cities. The equation of the DLNM model was as follows:

(2) $$Log\;E\left(Y_{k}\right)=\alpha +\sum cb\;\left(AP_{k},lag\right)+ns\left(time,\;\frac{7}{y}\right)+ns\left(AT,\;6\right)+ns\left(RH,\;6\right)+DOW+holiday$$

In the equation, ∑*cb* ( ) refers to the two-dimensional matrix of air pollutants and lag days; *AP_k_* refers to the mean air pollutants concentration on day *k*; and the other parts of the equation have the same meaning as above.

#### 2.3.3. Interaction Effect

The interaction analysis of drought and air pollutants consisted of two parts. At the first stage, a non-stratified model was established to explore the interaction effect of SPI-1 and air pollutants on pediatric URTI. If the interaction existed, non-parametric binary response models would be established to describe the spatial distribution characteristics of the effect of drought index and air pollutants on pediatric URTI. The model was fitted as follows:

(3) $$Log\left[E\left(Y_{k}\right)\right]=\alpha +TS\;\left(SPI-1_{k},\;AP\;lag02\right)+ns\left(time,\;\frac{7}{y}\right)+ns\left(AT,\;6\right)+ns\left(RH,\;6\right)+DOW+holiday$$

In the model, *TS* ( ) represented the thin-plate regression spline. Due to the lagged effects of air pollutants, we selected moving averages over lag 0 to 2 for air pollutant concentrations, which was the *AP lag*02 in the equation. The other parts of the equation showed the same meaning as above.

In phase two, the SPI-1 stratification model was established to explore whether the effect of air pollutants on pediatric URTI varied under different drought conditions. In addition, we stratified pediatric URTI visits by age and repeated the analyses in each subgroup. The differences in effects estimates between subgroups were tested by Z-test and the formula was as follows:

(4) $$Z=\frac{Q_{1}-Q_{2}}{\sqrt{SE_{1}^{2}+SE_{2}^{2}}}$$

*Z* is the z score; *Q*_1_ and *Q*_2_ are the estimated effects in two subgroups; and *SE*_1_ and *SE*_2_ are the respective standard errors of each group.

## 3. Results

### 3.1. Descriptive Statistics

There were 274,787 pediatric URTI outpatients under 6 included in this study from 1 January 2015 to 31 December 2018. On average, there were 125.3, 35.9, and 26.9 outpatient visits per day in large-scale hospitals in each city. Among them, outpatients aged 0 to 2 years old accounted for the largest proportion of outpatients. The characteristics of the average daily mean ambient temperature and relative humidity of each city during the study are shown in Table 1.

**Figure 1:**
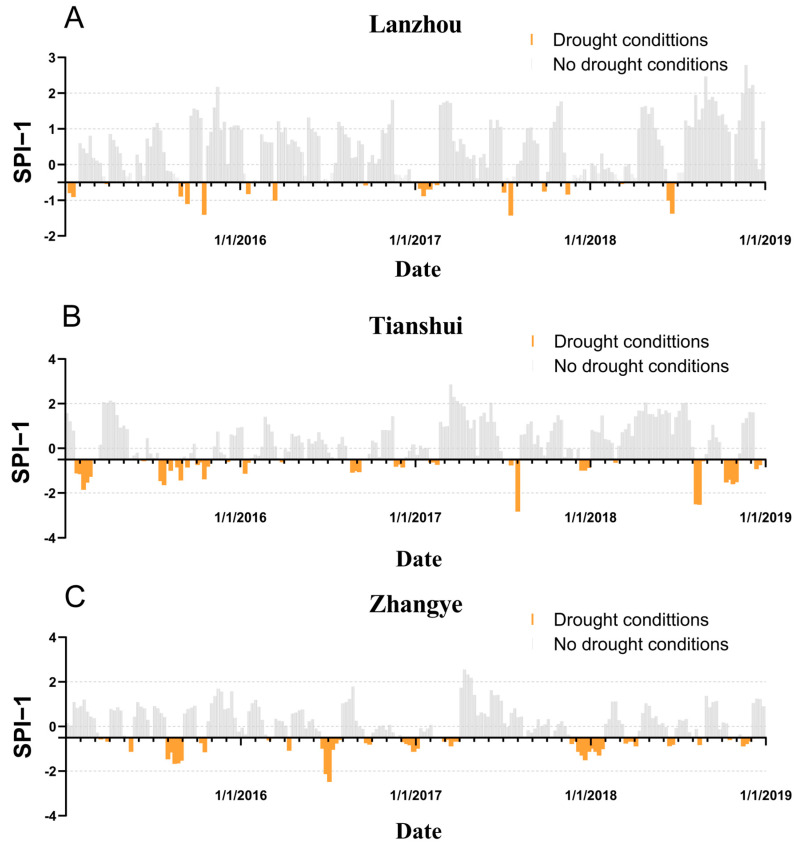
Drought characterization for Lanzhou (**A**), Tianshui (**B**), Zhangye (**C**) in Gansu province. Gray strips represent days with no drought conditions, and orange strips represent days with drought conditions.

As shown in Table 2, the mean concentration of PM_2.5_ and PM_10_ in three cities exceed the Grade I value of the China Ambient air quality standard (Table S1), and the maximum daily concentration for O_3_ exceeded the Grade II standard. Figure 1 displays the drought characterization for three cities during the study period. According to SPI-1, Lanzhou experienced drought about 15.06% of the days, Tianshui 25.94%, and Zhangye 24.52% (Table S2).

### 3.2. Correlation Analysis

Figures S1–S3 exhibit the Spearman correlation coefficient of meteorological factors, air pollutants, and SPI-1 in three cities. The correlation coefficients between SPI-1 and air pollutants were all less than 0.3 in three cities. The average daily temperature and humidity were negatively correlated with PM_2.5_, PM_10_, SO_2_, NO_2_, and CO, but positively correlated with O_3_.

### 3.3. Individual Effects

**Figure 2:**
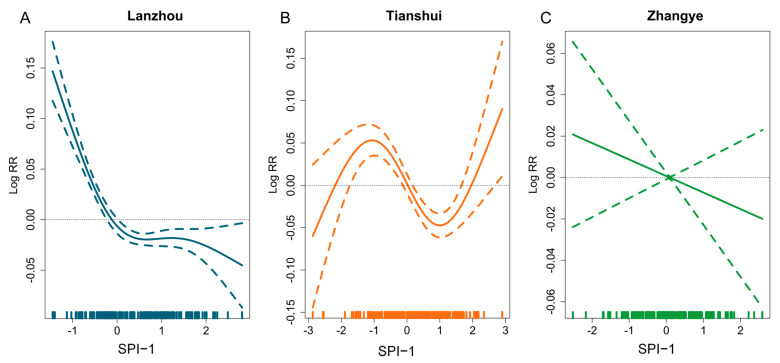
Dose–response associations between SPI-1 and pediatric URTI outpatient visits of Lanzhou (**A**), Tianshui (**B**), Zhangye (**C**) in Gansu province. The colorful solid line represent the dose-response curve in each city; the colorful dotted lines are the 95% confidence interval of Log RR in each city.

Figure 2 displays the exposure–response relationship between SPI-1 and daily pediatric URTI outpatient visits, which shows a downward trend. As SPI-1 increases, the number of daily outpatient visits decreases.

**Figure 3:**
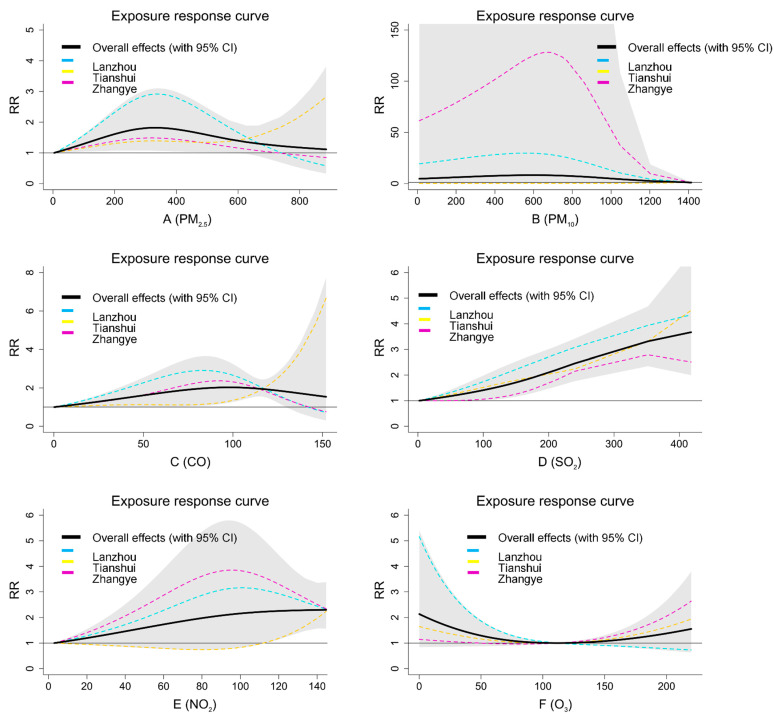
The overall and city-specific effects of air pollutants at lag 021 of daily outpatient visits for pediatric URTI in three cities.

The city-specific effects and overall estimates effects at lag 021 days are illustrated in Figure 3. There were significant associations between air pollutants and the number of daily pediatric URTI outpatient visits. PM_2.5_, PM_10_, CO, SO_2_, and NO_2_ had positive effects on the occurrence of pediatric URTI. The exposure–response curve of O_3_ is approximately a “V” shape. Figure S4 presented the overall estimated effects under different lag exposure periods (lag 03, lag 07, lag 014, and lag 021), which indicated that the longer the lag days, the stronger the estimated effects.

### 3.4. Interactive Effects

**Figure 4:**
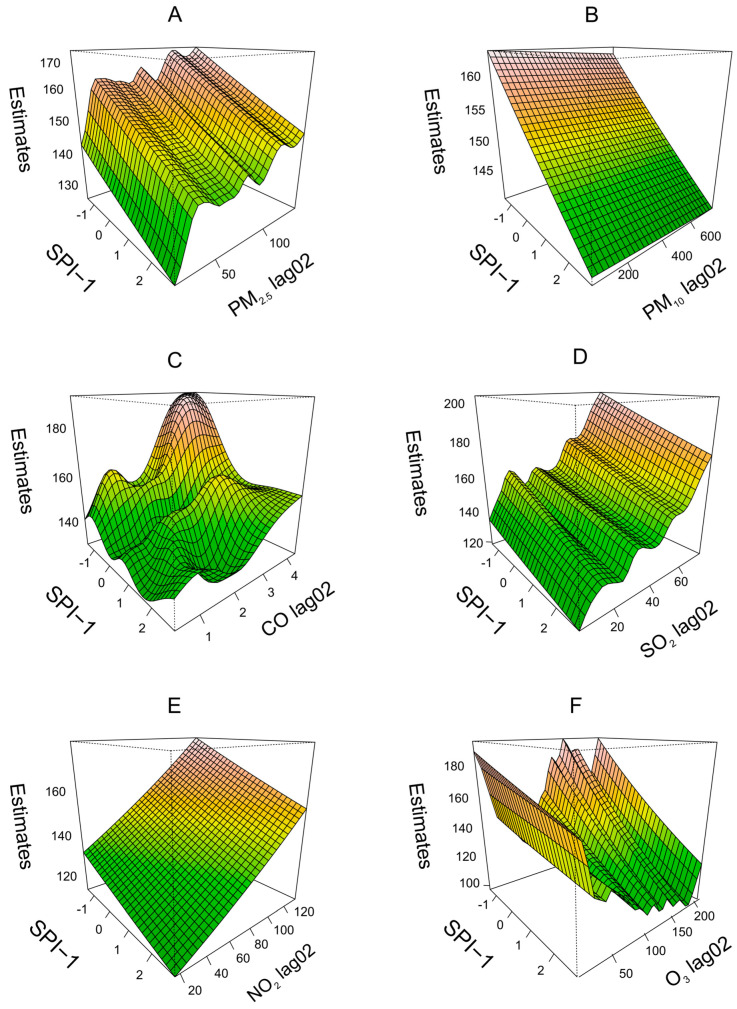
Bivariate response surface analysis of PM_2.5_ (**A**), PM_10_ (**B**), CO (**C**), SO_2_ (**D**), NO_2_ (**E**) and O_3_ (**F**), and SPI-1 in pediatric URTI in Lanzhou city.

Due to the lagged effect of pollutants on pediatric URTI outpatient visits, we established the bivariate response surface models with SPI-1 and lag 02 days for air pollutants. Figure 4 exhibits the spatial distribution of the interactive effects of SPI-1 and lag 02 for air pollutants on pediatric URTI outpatient visits in Lanzhou city (plots for other cities are available in Figures S5 and S6). The interactive effects of PM_2.5_, PM_10_, CO, NO_2_, SO_2_, O_3_, and SPI-1 on pediatric URTI were obvious in Lanzhou, especially at a high level of these air pollutants. In the case of PM_2.5_, when SPI-1 was lower, the higher concentration of PM_2.5_, the more outpatient visits to pediatric URTI. Highly polluted and drought environments had the most significant impact on upper respiratory infections in children.

To clarify whether the effect of air pollutants on pediatric URTI varied under different drought conditions, we classified the severity of the drought period into four grades (Table S1). The quantitative relationships between the different drought conditions and air pollutants on pediatric URTI outpatient visits are summarized in Table 3. For every 10 μm/m^3^ increase in daily PM_2.5_, PM_10_, and SO_2_ at moderate drought conditions, the outpatient visits increased by 6.6% (95%CI: 3.6%, 9.8%), 1.8% (95%CI: 0.9%, 2.7%), and 11.4% (95%CI: 3.3%, 20.1%) in Lanzhou city compared to no drought condition. With each 10 μg/m^3^ increase in daily PM_2.5_, PM_10_, SO_2_, and NO_2_ during severe drought conditions, the risks for outpatient visits increased by 4.2% (95%CI: 2.9%, 5.4%), 3.3% (95%CI: 2.5%, 4.1%), 3.1% (95%CI: 1.5%, 4.6%), and 10.3% (95%CI: 7.8%, 12.9%) in Tianshui city. The risks for outpatient visits increased by 3.4% (95%CI: 2.6%, 4.1%) for every 0.1 mg/m^3^ increase in CO in Tianshui city. For O_3_, we observed protective effects on pediatric URTI during severe drought conditions in Tianshui and Zhangye city, which were different from those in Lanzhou city. The RRs were 0.964 (95%CI: 0.954, 0.975) and 0.975 (95%CI: 0.952, 0.998) for every 10 μg/m^3^ increase in O_3_ in Tianshui and Zhangye city.

### 3.5. Subgroup Analysis

**Figure 5:**
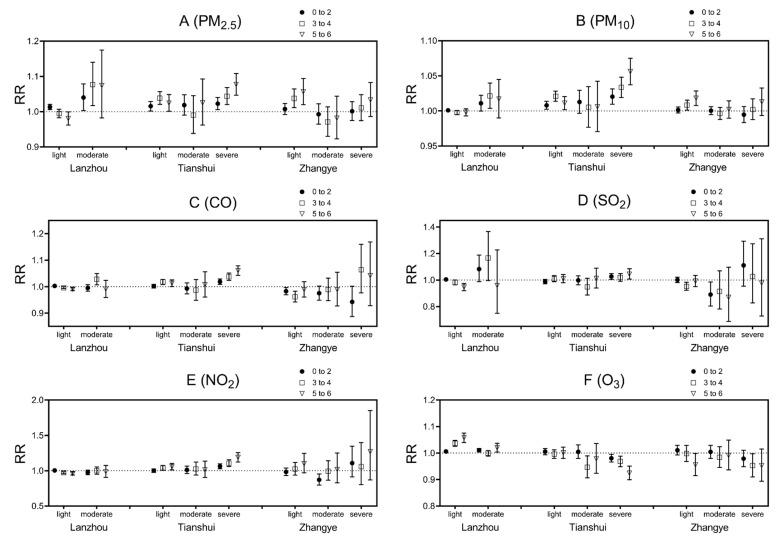
City-specific estimated relative risks (RR) and 95% confidence interval of daily outpatient visits for pediatric URTI under different age groups with a 10 μg/m^3^ (0.1 mg/m^3^ for CO) increase in daily mean air pollutant concentration by drought conditions.

Figure 5 displays the associations between the daily mean concentration of air pollutant concentrations and daily city-specific pediatric URTI outpatient visits in light, moderate, and severe drought conditions by age. The interactions between air pollutants and drought varied on air pollutants in different cities. With the increase in daily PM_2.5_, PM_10_, and NO_2_ at severe drought conditions, outpatient visits increased in all age groups compared with no drought conditions.

## 4. Discussion

We built different models based on time-series analyses to estimate the effect of drought and air pollutants on pediatric URTI outpatient visits in Gansu province and we found evidence of the interactive effect of drought and air pollutants on children’s URTI. We believe this evidence adds to the knowledge of the interactive effect of air pollutants and drought on respiratory health among children.

Abundant studies have investigated the effects of air pollutants on the health of children’s upper respiratory tract [36,39,40,41]. Research conducted in Hefei found that air pollutants were associated with an increased risk of pediatric URTI outpatient visits, and located that NO_2_ was the major air pollutant that affected URTI outpatients [36]. Similar results were found in the presence of air pollutants on pediatric URTI in our study. There were significant associations between air pollutants and the number of pediatric URTI visits in these three cities, and the lag effects of air pollutants were also observed. Ambient PM consists of solid and liquid particles suspended in the atmosphere, which can easily enter the respiratory system [42]. Particles with a smaller diameter can enter deeper and lead to respiratory infections by promoting inflammation, oxidative stress, and reducing the function of immune cells, especially antimicrobial function [43,44]. The cellular mechanism by other air pollutants also exerted similar adverse effects on organs [45].

SPI-1 was used as an indicator to assess drought in this study, which has been used in several similar studies [46,47,48]. According to the results obtained through GAM models, the number of daily outpatient visits decreased with the increase in SPI-1, which means the more severe the drought is, the higher the risk is in URTI of children. This is consistent with our previous study, which demonstrated that drought increased the adverse effects on all respiratory diseases [24]. In the Brazilian Amazon, a prior study based on children under 5 also found that drought conditions exacerbated the incidence of respiratory diseases [22]. Prolonged rainfall deficiency is the most important characteristic of drought, and leads to a dry environment which has adverse effects on respiratory function [49,50]. URTI is caused mainly by virus infection [32]. Recent studies indicated a correlation between the stability of winter viruses and influenza virus particles in a dry environment [51]. Drought affects the soil conditions and vegetational cover, which perturb the upward transmission of dust [52]. As desertification intensifies, desert dust and toxic chemicals attached to its surface can seriously affect the health of the upper respiratory tract [53]. Wildfires frequently occur during droughts and release large amounts of aerosols into the atmosphere, and air quality deteriorates [54].

Drought may increase the amount of particulate matter suspended in the air [9,55]. There was no strong correlation between SPI-1 and pollutants found through correlation analysis in this study. It might be because SPI-1 was calculated by monthly precipitation, so the correlations analysis might be disturbed. However, many studies support the fact that drought can lead to higher concentrations of environmental pollution and more pollutants entering the respiratory system and causing diseases [53,55,56,57]. The interactive effects of drought and air pollutants on pediatric URTI outpatient visits in Gansu province were found in this study, indicating that the highly polluted and arid environment had significant impacts on URTI in children.

By detecting the sputum of respiratory infections patients, an experiment found that air pollutants were significantly associated with multi-drug-resistant bacteria [58]. PM was rich in bacteria and viruses [59], and as the amount of air pollutants increased in drought conditions, the risk of UTRI could be intensified. Air would be heated and humidified after entering the nose [60,61], dry air would cause the nasal mucosa epithelium to dry out and stimulate the epithelial cells to release inflammatory mediators, and air pollutants can aggravate the inflammatory response of the respiratory tract [50]. The declined immune defense function of the respiratory tract can make viruses or bacteria that already exist in the upper respiratory tract or invade from the outside multiply rapidly and may cause or aggravate the URTI [62,63]. The interaction between drought and air pollutants may be reducing the defense function of the airway, leading to URTI in children. For O_3_, we observed protective effects on pediatric URTI during severe drought conditions. It was also found in another study that a high concentration of O_3_ had a certain protective effect against rotavirus infection [59]. O_3_ has been demonstrated to have broad-spectrum antimicrobial and antiviral activity [64]. However, since O_3_ is also one kind of irritant gas to the respiratory system, the results found in this study also need to be verified in further studies.

Although we provided evidence that there were significant associations between the occurrence of drought and URTI in children and the interactive effects of drought and air pollutants on URTI. There are some limitations in the study that should not be ignored. First, we only used average exposure levels due to the unavailability of personal environmental exposures, and this is an ecological study, and ecological errors cannot be avoided. Second, we use weekly SPI-1 to assume the same conditions at intervals every 7 days, which may lead to potential exposure misspecification. Third, we did not obtain the gender of all outpatients and were not available to discuss gender-specific relationships with drought and pollutants. Last but not least, our study only included three arid regions in northwest China, and regional discrepancies should be considered when conducting similar studies in other regions.

## 5. Conclusions

In this time-series study, we noticed that the occurrence of drought and air pollutants increased the number of URTI outpatient visits in children, and evidence of the interaction of drought and air pollutants on pediatric URTI was found. These results suggest that improving air quality and developing policies to control and prevent drought-related diseases would benefit children’s health.

## Figures and Tables

**Table 1:** Characteristics of the daily study population and meteorological factors.

Variable		City	Mean ± SD	Min	P_25_	P_50_	P_75_	Max
Outpatient	Visits	Lanzhou	125.3 ± 51.7	27	93	117	149	406
		Tianshui	35.9 ± 16.5	5	24	34	44	130
		Zhangye	26.9 ± 11.9	0	19	26	34	107
Age	0–2	Lanzhou	83.6 ± 33.0	16	62	81	102	243
		Tianshui	19.3 ± 8.1	2	13	18	24	56
		Zhangye	16.4 ± 7.3	0	11	16	21	50
	3–4	Lanzhou	30.1 ± 17.4	1	18	28	37	129
		Tianshui	10.7 ± 6.6	0	6	10	14	51
		Zhangye	6.2 ± 4.3	0	3	5	9	28
	5–6	Lanzhou	11.6 ± 8.5	0	6	10	14	67
		Tianshui	5.8 ± 4.7	0	3	5	7	42
		Zhangye	3.0 ± 2.7	0	1	2	4	32
Meteorology	Mean temperature (°C)	Lanzhou	11.5 ± 10.0	−12.4	2.3	12.7	19.9	30.4
		Tianshui	12.5 ± 9.3	−10.2	4.1	13.5	20.5	30.4
		Zhangye	8.8 ± 12.1	−22.4	−1.7	10.3	19.5	30.8
	Mean relative humidity (%)	Lanzhou	50.5 ± 15.0	16	39	50	61.5	94
		Tianshui	64.9 ± 12.4	21	56	65	74	97
		Zhangye	46.4 ± 16.4	11	35	45	57	100

SD: standard deviation; min: minimum; P_25_: 25th percentile; P_50_: 50th percentile; P_75_: 75th percentile; max: maximum.

**Table 2:** Descriptive statistics of daily air pollutant concentrations.

Variables	City	Mean ± SD	Min	P_25_	P_50_	P_75_	Max
PM_2.5_ (μg/m^3^)	Lanzhou	47.6 ± 24.6	6.5	30.9	41.0	57.0	205.7
	Tianshui	41.0 ± 28.2	4.0	22.0	32.0	52.0	200.0
	Zhangye	36.3 ± 28.3	3.0	19.0	30.0	46.0	418.0
PM_10_ (μg/m^3^)	Lanzhou	122.0 ± 94.2	21.1	73.5	103.0	141.9	1413.8
	Tianshui	83.7 ± 56.3	10.0	45.0	66.0	108.0	684.0
	Zhangye	90.9 ± 92.3	6.0	42.0	67.0	104.0	886.0
CO (mg/m^3^)	Lanzhou	1.3 ± 0.7	0.2	0.8	1.0	1.5	5.0
	Tianshui	0.9 ± 0.5	0.2	0.5	0.7	1.1	3.8
	Zhangye	0.6 ± 0.4	0.1	0.4	0.5	0.8	4.6
SO_2_ (μg/m^3^)	Lanzhou	20.5 ± 15.2	3.2	9.3	15.0	28.0	87.8
	Tianshui	24.8 ± 23.6	2.0	9.0	12.0	36.0	176.0
	Zhangye	23.2 ± 27.6	2.0	8.0	14.0	20.0	152.0
NO_2_ (μg/m^3^)	Lanzhou	54.4 ± 21.9	11.0	38.4	51.8	66.0	144.8
	Tianshui	35.6 ± 15.6	7.0	24.0	32.0	46.0	100.0
	Zhangye	21.0 ± 8.8	3.0	14.0	20.0	26.0	52.0
O_3_ (μg/m^3^)	Lanzhou	62.9 ± 38.9	0.7	36.4	58.6	80.4	220.0
	Tianshui	90.6 ± 34.3	4.0	65.0	91.0	116.0	210.0
	Zhangye	105.6 ± 27.6	38.0	85.0	105.0	124.0	229.0

SD: standard deviation; min: minimum; P_25_: 25th percentile; P_50_: 50th percentile; P_75_: 75th percentile; max: maximum.

**Table 3:** City-specific estimated relative risks (RR) and 95% confidence interval of daily outpatient visits for pediatric URTI with a 10 μg/m^3^ (0.1 mg/m^3^ for CO) increase in daily mean air pollutant concentration by drought conditions.

Air Pollutants	City	Drought Condition		
		Light Drought	Moderate Drought	Severe Drought
PM_2.5_	Lanzhou	1.006 (1.012, 1.000) *	1.066 (1.036, 1.098) ***	
	Tianshui	1.024 (1.014, 1.034) ***	1.019 (0.996, 1.043)	1.042 (1.029, 1.054) ***
	Zhangye	1.017 (1.005, 1.030) **	0.995 (0.974, 1.017)	1.014 (0.995, 1.033)
PM_10_	Lanzhou	1.000 (0.998, 1.001)	1.018 (1.009, 1.027) ***	
	Tianshui	1.013 (1.009, 1.017) ***	1.012 (0.998, 1.025)	1.033 (1.025, 1.041) ***
	Zhangye	1.004 (1.001, 1.008) *	1.002 (0.997, 1.006)	1.001 (0.994, 1.009)
CO	Lanzhou	1.000 (0.998, 1.002)	1.005 (0.995, 1.015)	
	Tianshui	1.008 (1.003, 1.014) **	0.997 (0.980, 1.014)	1.034 (1.026, 1.041) ***
	Zhangye	0.979 (0.969, 1.032)	0.979 (0.959, 1.000)	1.001 (0.958, 1.047)
SO_2_	Lanzhou	0.993 (0.984, 1.002)	1.114 (1.033, 1.201) **	
	Tianshui	0.999 (0.986, 1.011)	0.995 (0.968, 1.023)	1.031 (1.015, 1.046) ***
	Zhangye	0.987 (0.972, 1.002)	0.884 (0.818, 0.956) **	1.036 (0.926, 1.159)
NO_2_	Lanzhou	0.991 (0.985, 0.998) *	0.986 (0.960, 1.012)	
	Tianshui	1.021 (1.003, 1.039) *	1.014 (0.973, 1.057)	1.103 (1.078, 1.129) ***
	Zhangye	1.114 (0.965, 1.286)	0.917 (0.856, 0.983) *	1.114 (0.965, 1.286)
O_3_	Lanzhou	1.018 (1.013, 1.023) ***	1.008 (1.002, 1.013) **	
	Tianshui	1.002 (0.993, 1.010)	0.989 (0.969, 1.009)	0.964 (0.954, 0.975) ***
	Zhangye	1.007 (0.993, 1.021)	1.009 (0.990, 1.028)	0.975 (0.952, 0.998) *

Figures in bold are statistically significant. * *p* < 0.05, ** *p* < 0.01, and *** *p* < 0.001.

## Data Availability

The meteorological datasets and air pollutants datasets used and/or analyzed are available from the open access websites. The meteorological datasets from Lanzhou Meteorological Bureau. All data on outpatient visits were obtained from hospitals or health bureaus with their permission.

## Supplementary Materials

The following supporting information can be downloaded at: https://www.mdpi.com/article/10.3390/ijerph20031959/s1, Table S1: The ambient air pollutant concentration limits of China Ambient air quality standard (GB 3095-2012); Table S2: Grades of meteorological drought in three cities according to SPI-1 (GB/T 20481-2017); Figure S1: Pearson correlation between SPI-1 and air pollutants in Lanzhou; Figure S2: Pearson correlation between SPI-1 and air pollutants in Tianzhui; Figure S3: Pearson correlation between SPI-1 and air pollutants in Zhangye; Figure S4: The overall estimates effects under different lag exposure period; Figure S5: Bivariate response surface analysis of air pollutants and SPI-1 in child URTI in Tianshui city; Figure S6: Bivariate response surface analysis of air pollutants and SPI-1 in child URTI in Zhangye city.
