# Supplementary material for: The Interactive Effects between Drought and Air Pollutants on Children’s Upper Respiratory Tract Infection: A Time-Series Analysis in Gansu, China
Source: Int J Environ Res Public Health. 2023 Jan 20;20(3):1959. doi: 10.3390/ijerph20031959 (PMC9915313; doi:10.3390/ijerph20031959)
Supplement: Supplementary file 1 [file ijerph-20-01959-s001.zip › ijerph-2142154-supplementary.pdf]

Supplementary Material

Table S1 displays ambient air quality standards in China.

Table S2 summarizes the grades of drought in three cities according to SPI-1. Surface water resources are sufficient to meet production and living needs during no drought periods. Light drought periods, when the surface air is dry and soil moisture is mildly insufficient but has little impact on production and life. In times of moderate drought, soil moisture is insufficient and water resources are scarce, affecting production and livelihood. Severe drought periods, with severe soil moisture deficits and water shortages, have a heavy impact on production and livelihood.

Figures S1-S3 exhibit the Pearson correlation between SPI-1 and air pollutants in three cities.

Figure S4 shows the overall estimates effects under different lag exposure period.

Figures S5 and S6 exhibit the bivariate response surface analysis of air pollutants and SPI-1 in child URTI in Tianshui and Zhangye city.

Table S1. The ambient air pollutant concentration limits of China Ambient air quality standard (GB 3095-2012).

Varibles	Average duration	Concentration limit	
		Grade I	Grade II
$PM_{2.5}$ (μg/m^3)	24-hour mean	35	75
	Annual mean	15	35
PM_{10} (μg/m^3)	24-hour mean	50	150
	Annual mean	40	70
CO (mg/m^3)	24-hour mean	4	4
	1-hour mean	10	10
SO_2 (μg/m^3)	24-hour mean	50	150
	1-hour mean	150	500
	Annual mean	20	60
NO_2 (μg/m^3)	24-hour mean	80	80
	1-hour mean	200	200
	Annual mean	40	40
O_3 (μg/m^3)	8-hour mean	100	160
	1-hour mean	160	200

Table S2. Grades of meteorological drought in three cities according to SPI-1 (GB/T 20481-2017).

Grades of drought	Number of drought days during the study period		
	Lanzhou	Tianhui	Zhangye
No drought (-0.5＜SPI-1)	1241 (84.94%)	1082 (74.06%)	1059 (72.48%)
Light drought (-1.0＜SPI-1≤-0.5)	178 (12.18%)	218 (14.92%)	245 (16.77%)
Moderate drought (-1.5＜SPI-1≤-1.0)	42 (2.87%)	63 (4.31%)	108 (7.39%)
Severe drought (SPI-1≤-1.5)	0 (0.00%)	98 (6.71%)	49 (3.35%)

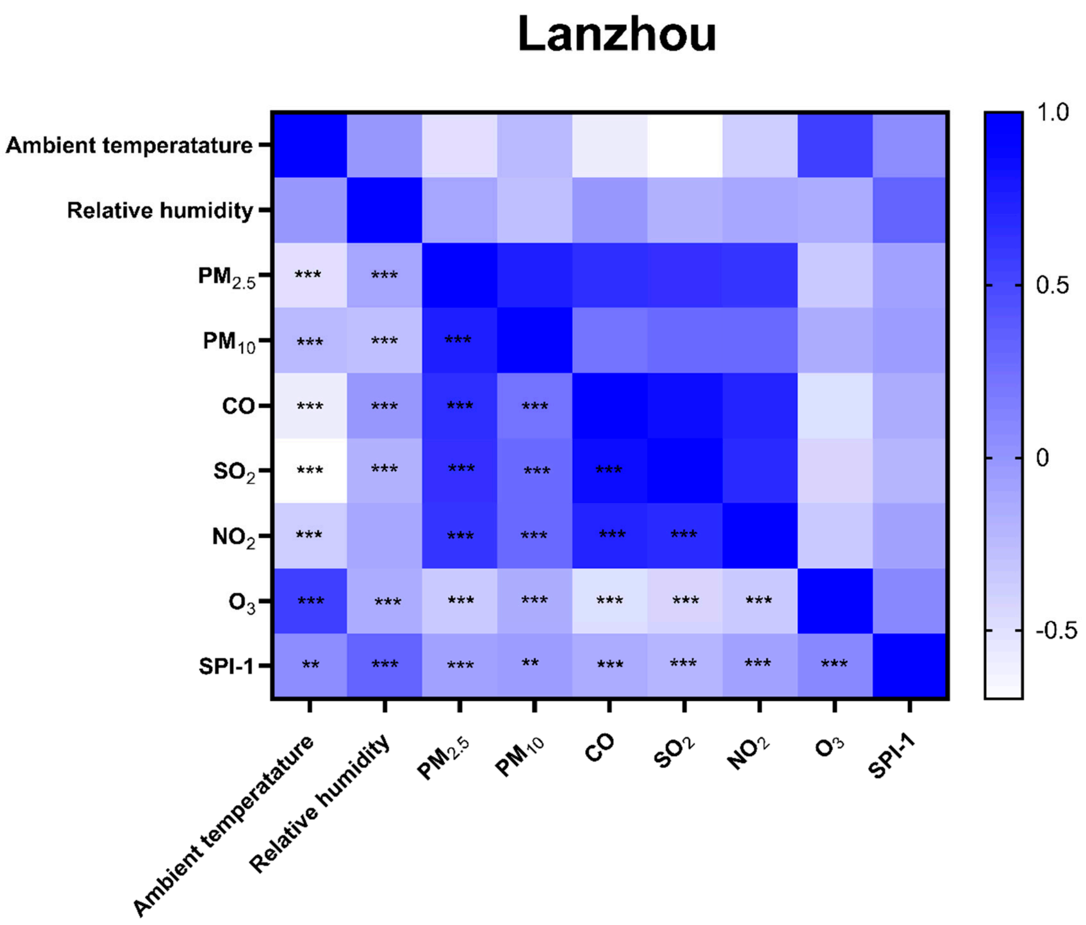

Figure S1. Pearson correlation between SPI-1 and air pollutants in Lanzhou. SPI in the figure stands for SPI-1. * $p<0.05$, ** $p<0.01$, and *** $p<0.001$.

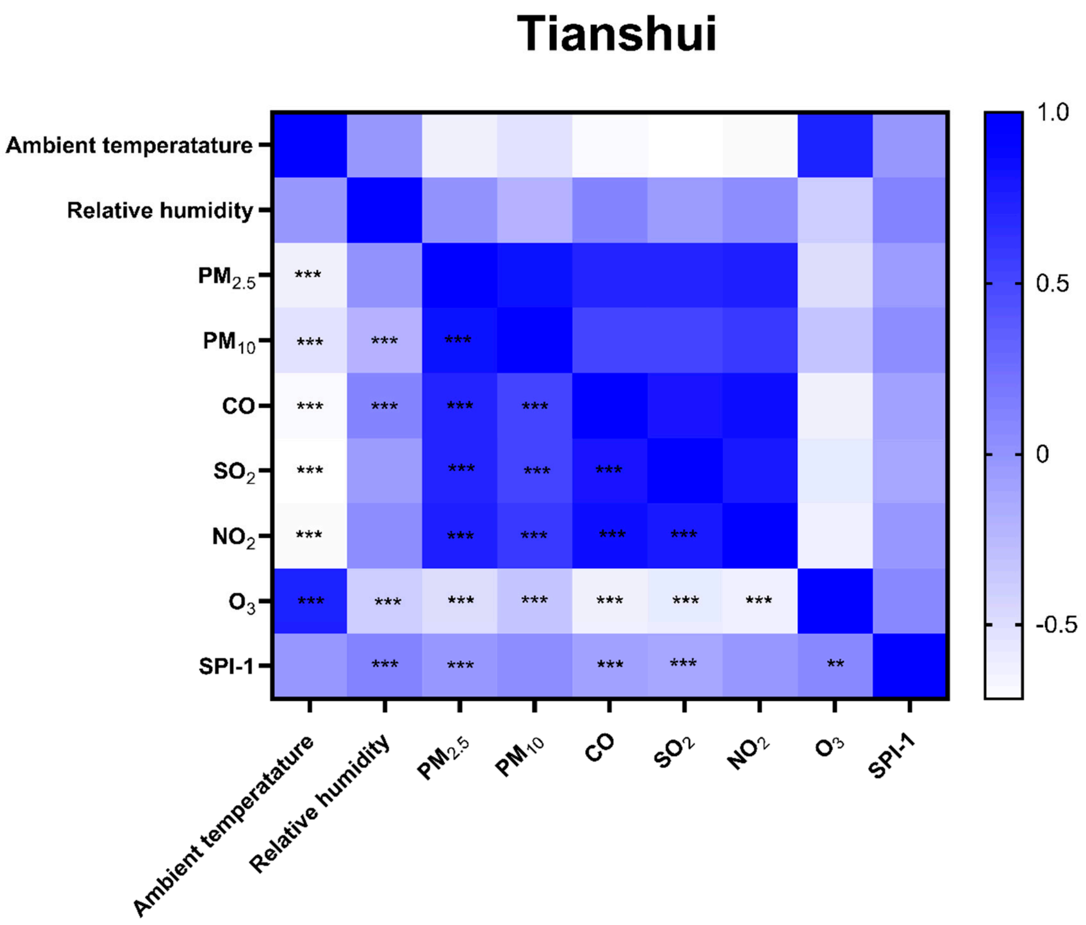

Figure S2. Pearson correlation between SPI-1 and air pollutants in Tianzhui. SPI in the figure stands for SPI-1. * $p<0.05$, ** $p<0.01$, and *** $p<0.001$.

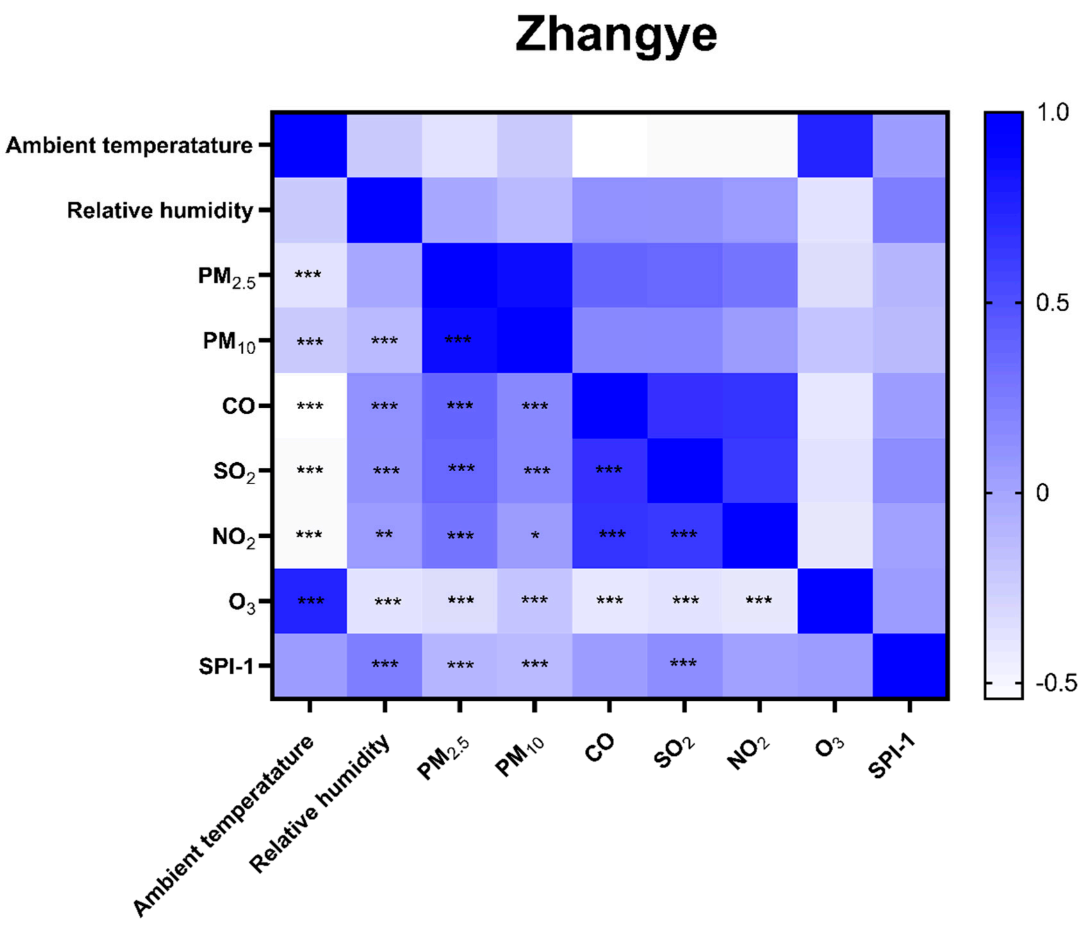

Figure S3. Pearson correlation between SPI-1 and air pollutants in Zhangye. SPI in the figure stands for SPI-1. * $p<0.05$, ** $p<0.01$, and *** $p<0.001$.

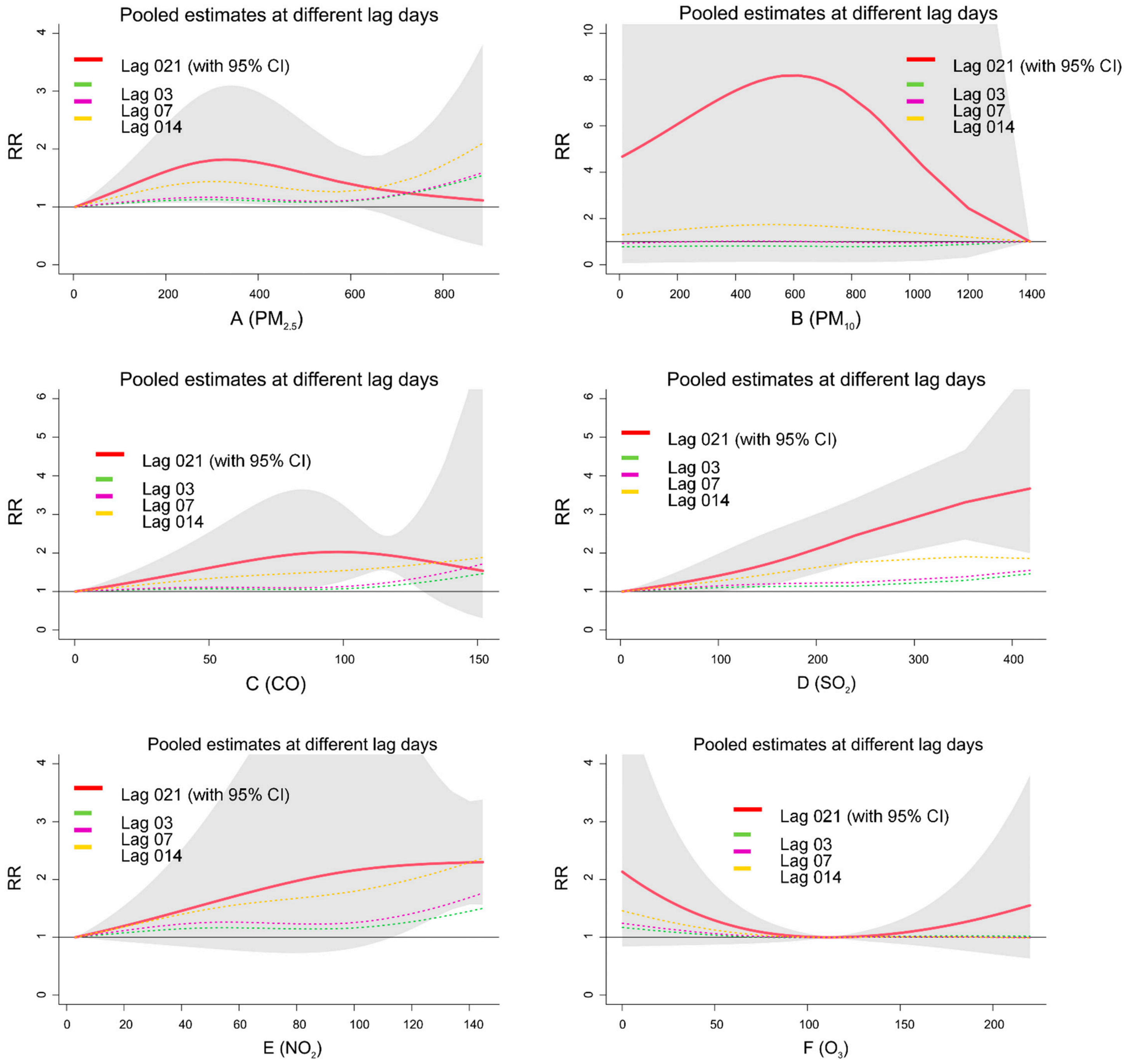

Figure S4. The overall estimates effects under different lag exposure period (lag 03, lag 07, lag 014, and lag 021).

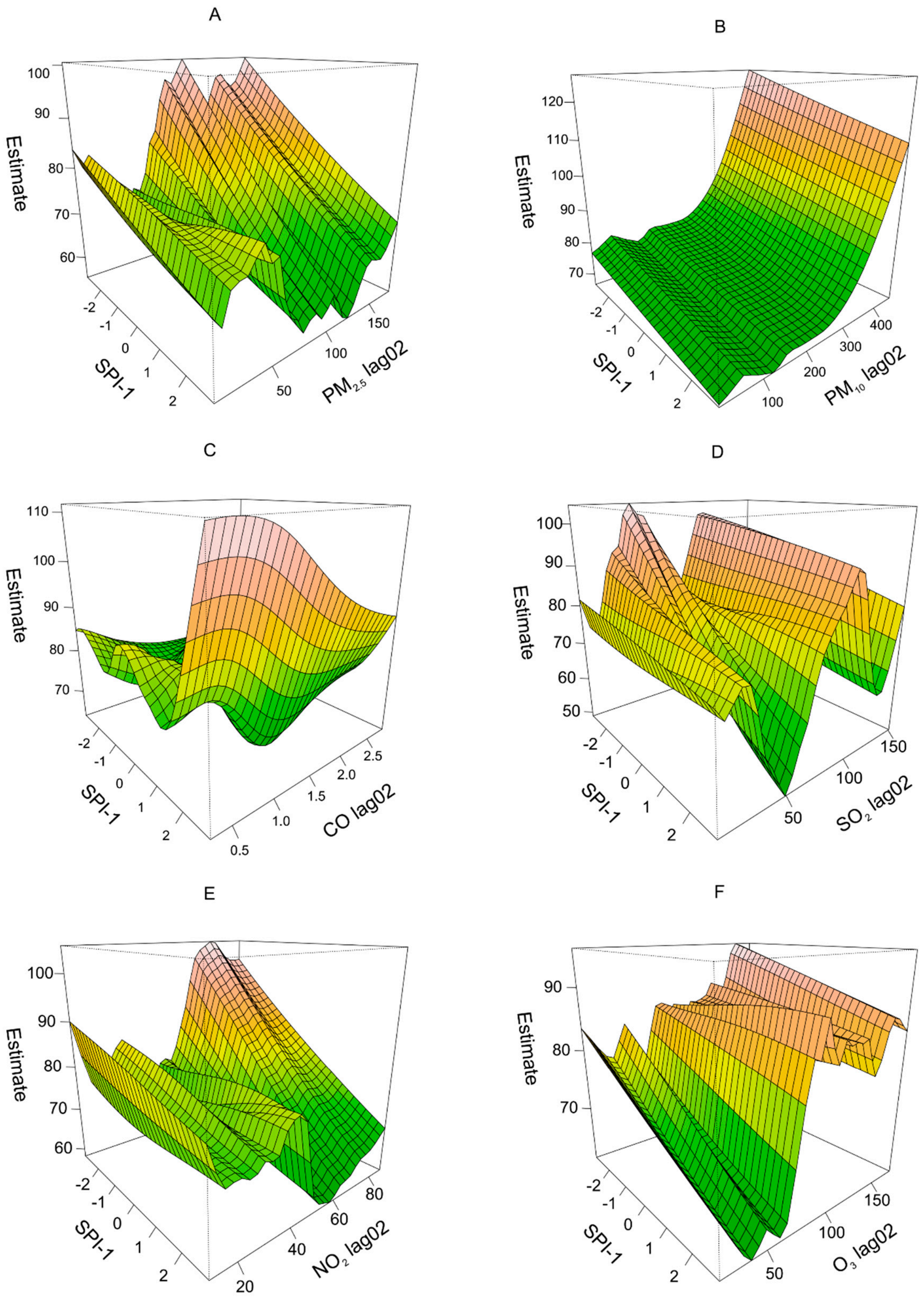

Figure S5. Bivariate response surface analysis of air pollutants and SPI-1 in child URTI in Tianshui city.

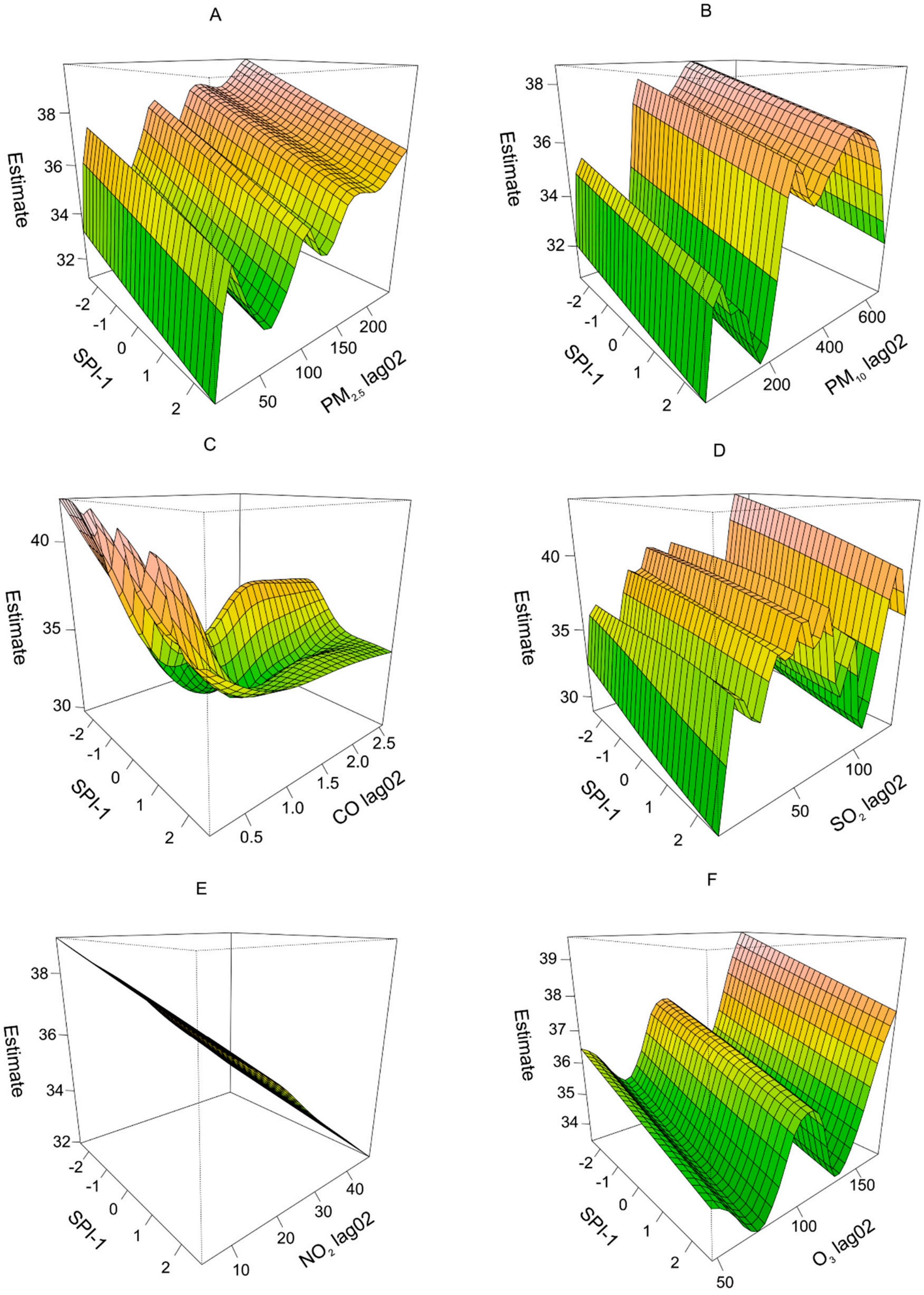

Figure S6. Bivariate response surface analysis of air pollutants and SPI-1 in child URTI in Zhangye city.
